# The prevalence of glucose-6-phosphate dehydrogenase deficiency in the Cape Verdean population in the context of malaria elimination

**DOI:** 10.1371/journal.pone.0229574

**Published:** 2020-03-16

**Authors:** Adilson José DePina, Cecílio Mendes Pires, Alex Jailson Barbosa Andrade, Abdoulaye Kane Dia, António Lima Moreira, Maria Celina Moreira Ferreira, Artur Jorge Correia, Ousmane Faye, Ibrahima Seck, El Hadji Amadou Niang

**Affiliations:** 1 Programa Eliminação do Paludismo, CCS-SIDA, Ministério da Saúde e da Segurança Social, Praia, Cabo Verde; 2 Ecole Doctorale des Sciences de la Vie, de la Santé et de l´Environnement (ED-SEV), Université Cheikh Anta Diop (UCAD) de Dakar, Dakar, Sénégal; 3 Laboratório de Análises Clínicas, Hospital Regional de Santiago Norte, Assomada, Cabo Verde; 4 Instituto Nacional de Gestão do Território, Praia, Cabo Verde; 5 Laboratoire d’Ecologie Vectorielle et Parasitaire, Faculté des Sciences et Techniques, Université Cheikh Anta Diop (UCAD) de Dakar, Dakar, Sénégal; 6 Programa Nacional de Luta contra o Paludismo, Ministério da Saúde e da Segurança Social, Praia, Cabo Verde; 7 Unidade Seguimento e Avaliação, CCS-SIDA, Ministério da Saúde e da Segurança Social, Praia, Cabo Verde; 8 Direcção Nacional da Saúde, Ministério da Saúde, Praia, Cabo Verde; 9 Institut de Santé et Développement, Université Cheikh Anta Diop (UCAD) de Dakar, Dakar, Sénégal; Mahidol Oxford Clinical Research Unitl (MORU), THAILAND

## Abstract

Cabo Verde aims to eliminate malaria by 2020. In the country, *Plasmodium falciparum* had been the main parasite responsible for indigenous cases and primaquine is the first line treatment of cases and for radical cure. However, the lack of knowledge of the national prevalence of glucose-6-phosphate dehydrogenase (G6PD) deficiency may be one of the constraints to the malaria elimination process. Hence, this first study determines the prevalence of G6PD deficiency (G6PDd) in the archipelago. Blood samples were collected from patients who voluntarily agreed to participate in the study, in the health facilities of eight municipalities on four islands, tested with G6PD CareStart ^™^ deficiency Rapid Diagnosis Test (RDT). All subjects found to be G6PDd by RDT then underwent enzyme quantification by spectrophotometry. Descriptive statistics and inferences were done using SPSS 22.0 software. A total of 5.062 blood samples were collected, in majority from female patients (78.0%) and in Praia (35.6%). The RDT revealed the prevalence of G6PD deficiency in 2.5% (125/5062) of the general population, being higher in males (5.6%) than in females (1,6%). The highest G6PDd prevalence was recorded in São Filipe, Fogo, (5.4%), while in Boavista no case was detected. The G6PDd activity quantification shown a higher number of partially deficient and deficient males (respectively n = 26 and n = 22) compared to females (respectively n = 18 and n = 7), but more normal females (n = 35) than males (n = 11). According to the WHO classification, most of the G6PDd cases belongs to the class V (34.5%), while the Classes II and I were the less represented with respectively 5.8% and zero cases. This study in Cabo Verde determined the G6PDd prevalence in the population, relatively low compared to other African countries. Further studies are needed to characterize and genotyping the G6PD variants in the country.

## Introduction

Malaria remains one of the major public health problem worldwide, despite the unprecedented gains made so far, with the reduction of the morbidity and mortality thus allowing its elimination in some countries [1,2]. Based on the successes recorded in several countries, the World Health Organisation (WHO) has identified in 2016 at least 16 countries with the potential to eliminate malaria by 2020 [3]. Among the selected candidates, Cabo Verde is carrying out a pre-elimination process with the ultimate goal of elimination.

Historically, malaria has been the first vector-borne disease in Cabo Verde since the 16^th^ century during the settlement of the islands, and with some efforts, the disease has been eliminated twice from the archipelago. In the last years, the malaria epidemiological situation has become unstable with the sporadic seasonal transmission, and local transmission exclusively occurring in Santiago and Boavista islands [4,5].

Despite the low malaria incidence, the archipelago is still prone to outbreaks, as the most recent one recorded in 2017 with 423 indigenous cases [5]. This shows the country’s vulnerability to malaria, which constitutes a big challenge for the elimination goal. Moreover, this stressed the urgent need to readapt the malaria control interventions as well as the responses to outbreaks to get the country back on its elimination path, resulting in zero autochthonous cases in 2018. However, to consolidate the gains and achieve malaria elimination in Cabo Verde, several actions must be reinforced and adapted to the context. One action concerns the treatment and follow-up of the confirmed malaria cases. According to the Cape Verdean National Malaria Control Protocol [6], all the confirmed *Plasmodium falciparum* malaria cases are treated with Artemether + Lumefantrine (AL) and a single dose of primaquine (0.25 mg/kg body weight) in the first day of treatment. While all the *P*. *vivax* and *P*. *ovale* malaria cases receive the primaquine with the aim to eliminate the hypnozoites. The schizontocidal treatment for non–falciparum malaria is done with the AL or Artesunate + Amodiaquine, following the WHO guidelines [7]. Nevertheless, this use of primaquine is carried out regardless the knowledge of the frequency of the Glucose-6-phosphate-dehydrogenase (G6PD) deficiency among the target population, which endangers all the patients harbouring this deficiency with the risk of haemolysis [8–12].

The G6PD deficiency (G6PDd) is a X-chromosomally transmitted disorder, affecting both men and women, and individuals with G6PD deficiency may develop potentially fatal anaemia during food-induced oxidative stress, medication or infection by microorganisms [13]. The G6PD deficiency affects about 400 million persons worldwide [8], is mainly associated with some ethnic groups. The highest prevalence of the G6PDd, between 15% and 26%, are recorded in Africa, Asia, the Mediterranean and the Middle East [14–16].

Studies carried out have identified so far approximately 220 genetic variants causing clinical deficiency linked to the G6PDd [17–20]. The geographical distribution of these deficiency alleles is extensive and correlated with populations exposed historically to endemic malaria [8,16]. In addition, few studies support that the G6PD deficiency is an adaptative response providing protection against some severe forms of *P*. *falciparum* malaria [20–22].

The high prevalence of G6PDd occurs in Sub-Saharan Africa [8, 17, 22] and Western Africa, the G6PDd range between 10 and 20% or more [16, 23]. Taking into account this distribution, beside that the Cape Verdean population consists of different ethnic origins with a diversified degree of racial miscegenation, the lack of national records on the G6PDd while using the primaquine, stresses the urgent need to determine the prevalence of G6PDd in the country population to better support decision-making and correct management of malaria cases in elimination process in the country.

## Methods

### Study design

This was a cross sectional prevalence study to determine the G6PDd prevalence in Cape Verde conducted from September to December 2018 in four out of the nine inhabited islands of the country. During the study period, a total of 5062 volunteers were enrolled to estimate the prevalence of the G6PD deficiency. The technical staff consisted of 50 volunteers, including laboratory technicians, mobilizers and coordination members were involved in collection and analysis of this study.

### Study area

**Fig 1:**
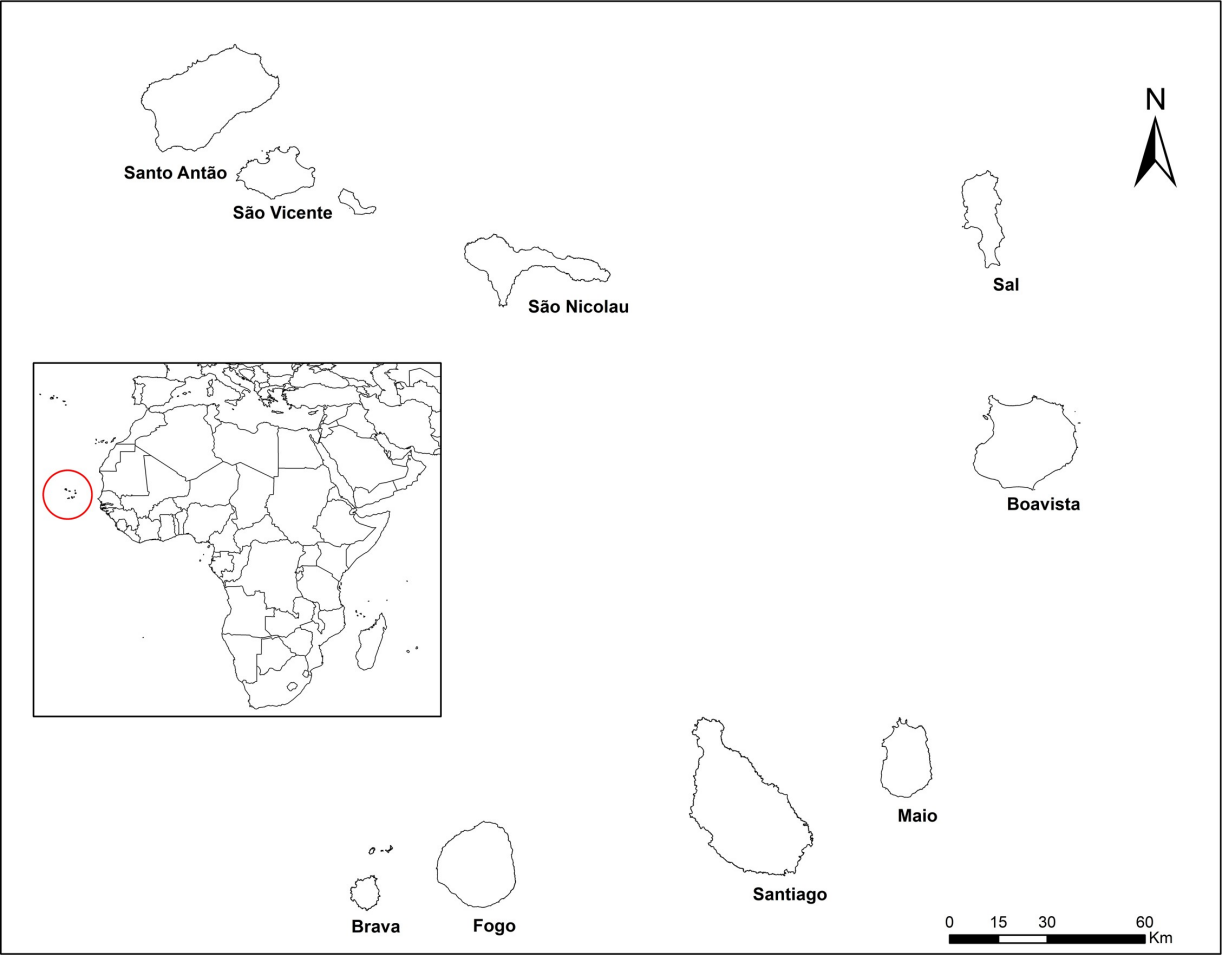
Localization of Cabo Verde, study area.

The study was carried out in the archipelago of Cape Verde, which consists of ten islands (Fig 1). The country covers an area of 4033 Km^2^ with about half millions of residents. The study was conducted on four islands and 8 municipalities based on the risk of malaria in 2017. On the one hand, the islands of Santiago (in Praia, Santa Catarina, Santa Cruz, São Miguel and Tarrafal municipalities) and Boavista where indigenous malaria cases were reported the last year. While the islands of Fogo (São Filipe) and São Vicente were selected as areas without any local cases during the last century. Noteworthy, Boavista and São Vicente islands consist each of only one municipality of the same name, where the study was conducted.

### Study population

Consenting informed volunteers residing in the four selected islands were included in the study. The sample size for each study population was determined using data of the last Cape Verdean population census from the National Statistics Institute [24], which estimated in 2018 national population to 544081 inhabitants. Samples were selected based on the population proportion in each site and with absolute precision (d) of 2.5%, a confidence level of 95% (z = 1.96) and p of 0.5.

Sample collection was carried randomly from patients attending the health structures in Santiago island, namely, Praia, Santa Catarina, Santa Cruz, São Miguel and Tarrafal municipalities, in Boavista, São Filipe in Fogo and in São Vicente. Individuals of both sexes aged of at least 3 years, who agreed personally, or on the behalf of the authorized person, to participate in the study were enrolled then screened using the G6PD deficiency CareStart ^™^ RDTs of (Access Bio, New Jersey, USA), following the manufacturer's instructions. The test has a sensitivity (95% CL) 100% and specificity 97%. Approximately 5 ml of venous blood samples were collected by properly trained laboratory technicians from all G6PD deficient volunteers using sterile venepuncture device and stored in individual tubes containing an Ethylene Diamine Tetra-Acetic Acid (EDTA). Samples were then sent to and stored at +4°C in the Laboratory of Clinical Analyses of the Regional Hospital of Santiago Norte, in Santiago island, for the further analysis to confirm and quantify G6PD the deficiency. People refusing to sign the terms of the consent form or withdrawing the engagement were excluded. Three individuals from which insufficient blood samples were collected for the qualitative and quantitative analysis of the G6PD and samples which did not meet the qualities for the subsequent processessing were excluded.

In addition to the blood samples collected from each participant, supplement information including the sex, age, residence, citizenship, education, marital status, were also collected, using a pre-conceived data collection sheet.

### Laboratory analysis

#### Qualitative analysis of the G6PD deficiency

Labelled EDTA-stored venous blood samples were received and individually verified by the quality controller and properly stored in the laboratory at +4°C and analysed within the next three days upon reception. The same G6PD deficiency CareStart ^™^ RDTs of (Access Bio, New Jersey, USA) were used to confirm the previous results. To crosscheck the enzyme sensitivity, all the cases classified as G6PD deficient or indeterminate were confirmed by quantitative enzymatic analysis spectrophotometry using the RANDOX G-6-PDH KIT, performed on the AU480 automatic analyser (Beckman, Inc.), with temperature controlled, at 37ºC.

#### Haematocrit of the whole blood samples

The Unicell DxH 800 Beck Coulter was used to count the Red blood cells of each enrolled subject according to their G6PD status as G6PD deficient or intermediate. Samples collected from the Santa Catarina and São Miguel Health centres, both in Santiago Island, were analysed within 24 hours after the collection. While those from the other municipalities of Santiago as well as from the other islands were transported refrigerated (+4°C) and processed within 72 hours in the Santiago Norte Regional Hospital Laboratory. All the equipment were daily controlled and calibrated before carrying out the tests.

#### Quantitative analysis of the G6PD deficiency

The enzymatic activity the G6PD deficiency was quantified on the Beckman Coulter AU480 equipment from the whole blood of each G6PD deficient volunteers using the RANDOX G-6-PDH reagent kit, following the manufacturer's instructions. The lyophilized controls were diluted with 0.5 ml of distilled water (Cat.NO.PD 2617 and PD 2618) then stored at +4°C for use within 120 hours. The control samples were used according to the manufacturer’s criteria to enzymatic each test and control the quality of G6PD enzyme activity; with the values 1298mU/ml and 214mU/ml corresponding respectively to a normal and poor enzymatic activity.

The results of the quantitative analysis of the G6PD deficiency were generated using a formula pre-incorporated in the software associated with the automatic analyser AU480. The results of the G6PD activity were interpreted according to the manufacturer's guidelines, as i) deficient for an activity <2.5 U / g Hb; ii) partially deficient for activity between 2.5 to 7.8 U / g Hb; and iii) normal for an activity ≥ 7.9 U / g Hb. The observed G6PD variants were also classified according to the WHO guideline [25].

### Statistical analysis

All the demographic data of the volunteers (age, gender, ethnic group and place of residence), and the G6PDd test results were recorded in Microsoft Excel database and analysed using the SPSS 22.0 software version. Descriptive statistics and inferences to verify the association between independent variables and dependent variables were performed using the chi-square test. All variables were inserted into the binary logistic model to evaluate the adjusted odds ratio. Statistical significance was set at p < 0.05.

### Ethical approval

The study was approved by the National Committee for Ethics in Research for Health, of the Ministry of Health and Social Security, during its 863^rd^ Ordinary Meeting, held on September 27, 2018, according to the Resolution No. 53/2018.

Children and minors were included in the study after obtaining the signed authorization from parents or the legal guardian. Participation in the study was free and on a voluntary basis with a signed informed consent agreement from each participant or the legal guardian for a minor. All the individuals were recruited among those present within the health structures (laboratories, hospitals, health centres and health delegation) of the selected study sites during the study period. Potential participants present at the health structure received full details about the project objectives and the study protocol. All participants were free to leave the study or consult the information at any time point of the study.

Each volunteer was provided with an individual consent form for signing. Then a socio-epidemiological questionnaire was applied to each of the interviewees, using an accessible language.

## Results

### Sociodemographic characteristics of the study populations

A total of 5062 blood samples were collected from the eight municipalities of four Cape Verdean islands (Table 1). The majority of the samples were collected from Praia (35.6%, n = 1802), the capital of the country (Table 1). Almost all the participants were Cape Verdean (97.93%). Overall, the study population consisted mainly of females (78.03%, n = 3950). Over 53.56% of the participants were under the age of 35 years, with the age interval of 20–35 years being the most represented one (35.24%). Most of the participants had achieved at least the secondary educational level (40.87%) followed by the basic educational level (35.72%). The study population of study was composed mostly by single individuals (50.65%), cohabiting couples (30.60%), and the other 18.75% married, divorced, or widowed.

**Table 1:** Sociodemographic characteristics of study participants.

Categories	Tarrafal	Santa Cruz	São Miguel	Santa Catarina	Praia	São Vicente	São Filipe	Boavista	TOTAL (n)	%
**Sex**										
Female	231	427	202	459	1 391	721	439	80	3 950	78.03
Male	46	101	46	91	411	265	132	20	1 112	21.97
Total	277	528	248	550	1 802	986	571	100	5 062	
%	5.47	10.43	4.90	10.87	35.60	19.48	11.28	1.98	100.00	
**Age groups**										
0–4	8	23	2	9	45	5	17	0	109	2.15
5–9	23	59	1	22	90	24	27	1	247	4.88
10–14	5	49	3	28	85	21	26	1	218	4.31
15–19	16	30	26	54	135	48	38	6	353	6.97
20–24	23	37	26	67	208	96	60	17	534	10.55
25–29	39	39	30	71	254	98	79	20	630	12.45
30–34	39	43	27	42	266	100	79	24	620	12.25
35–39	16	39	19	43	181	94	78	12	482	9.52
40–44	16	35	22	24	128	86	44	8	363	7.17
45–49	17	25	12	26	74	82	26	2	264	5.22
50–54	23	38	19	37	99	85	20	2	323	6.38
55–59	13	35	15	40	96	70	32	4	305	6.03
60–64	17	28	19	30	64	63	23	2	246	4.86
+65	22	48	27	57	77	114	22	1	368	7.27
**Educational level**										
None	37	82	39	92	116	127	56	3	552	10.90
Pre-Primary	7	34	2	9	52	9	3	0	116	2.29
Basic	116	234	91	165	606	345	219	32	1 808	35.72
Secondary	101	154	87	243	790	390	247	57	2 069	40.87
Higher	16	24	29	41	238	115	46	8	517	10.21
**Marital Status**										
Married	50	103	52	92	224	136	101	11	769	15.19
Divorced	0	0	0	1	16	22	10	0	49	0.97
Single	147	314	141	353	870	432	260	47	2 564	50.65
Union of fact	63	97	42	77	668	367	193	42	1 549	30.60
Widowed	17	14	13	27	24	29	7		131	2.59
**Nationality**										
Cape Verdean	272	514	247	542	1742	984	566	90	4 957	97.93
Sao Tome and Principe	2	13	0	1	17	2	0	1	36	0.71
Guinea Bissau	1	0	0	1	14	0	1	8	25	0.49
Angolan	1	1	0	2	7	0	2	1	14	0.28
Senegalese	1	0	0	3	6	0	0	0	10	0.20
Portuguese	0	0	0	0	6	0	0	0	6	0.12
Others	0	0	1	1	10	0	2	0	14	0.28
**TOTAL**	**277**	**528**	**248**	**550**	**1 802**	**986**	**571**	**100**	**5 062**	**100.00**

### Prevalence and distribution of G6PD deficiency

**Fig 2:**
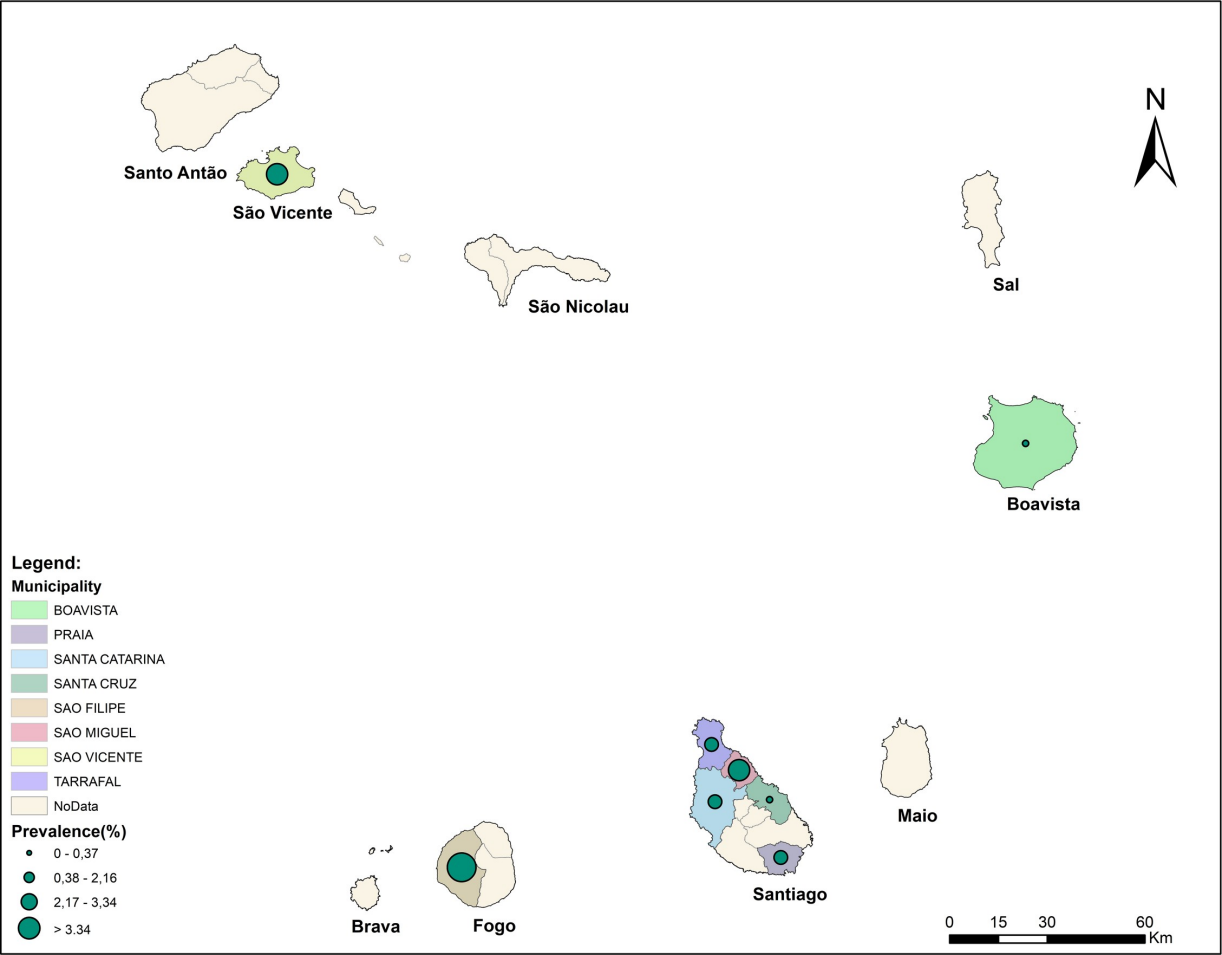
Prevalence of the G6PDd among the Cape Verdean population by municipalities.

Overall, of the 5062 samples collected, 125 (2.5%) was G6PD deficient result RDT. No G6PD deficiency was found in Boavista, which prevalence varied spatially between other islands and municipalities. Between islands, it was the highest in Fogo (5.4%) where the study was conducted in only one municipality (namely São Filipe), followed by the Island of São Vicente (3.3%). The overall G6PDd prevalence was 1.7% in the island of Santiago, being the highest in the municipality of São Miguel (3.3%) and the lowest in Santa Cruz (0.4%) (Fig 2). In the same island, Praia came at second position with 2.16%. Noteworthy, Santiago was the only study island of Cabo Verde where indigenous malaria cases were reported during the last three years [4,5], stressing the interest of such study in the different municipalities of the island.

The prevalence of G6PDd in the Cape Verdean population was higher in males (5.7%) than females (1.6%) (Table 2). One indeterminated case was found in a female individual.

**Table 2:** Prevalence of G6PDd by sex in Cape Verde.

Sex	RDT Carestart		Total	Percentage (%) of positives
	Negative	Positive		
Female	3 887	61	3 949	1.6%
Male	1 048	63	1 111	5.7%
**Total**	**4 936**	**124**	**5 062**	**2.5%**

#### Quantitative analysis of the enzymatic activities of the G6PD deficiency

After the confirmation of the G6PDd obtained from Carestart^TM^ RDT tests, analyses were performed to quantify the deficiency. Therefore, 119 G6PD deficient samples out of the 125 samples tested, were subsequently processed and quantified using the Randox G-6-PDH kit, adapted to the Beckman Coulter AU480. Six samples, one undetermined case and 5 cases of enrolled patients who left the study after the RDT tests, were not processed.

**Fig 3:**
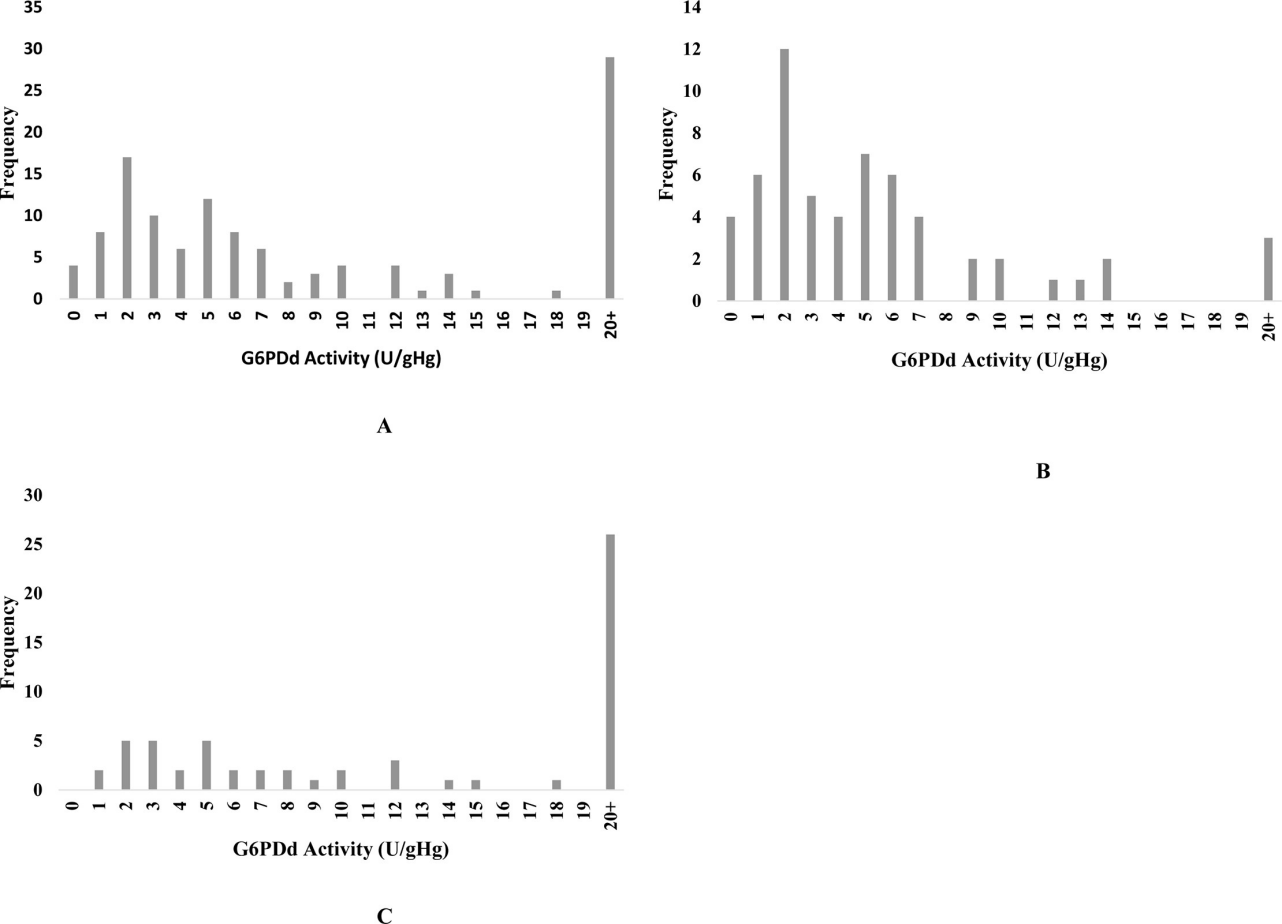
Distribuition of the G6PD activity in Cabo Verde, in according to gender. A—Distribuition in the general population. B in the male population and C in the female population.

The quantification of G6PDd positives samples, after the CareStart ^™^ RDTs, resulted in different results between male and female population (Fig 3). The activity values with more normal cases was observed in female (35 cases) than in male (11) population. The intermediate and deficient cases were more in male than females (26 to 18 and 22 to 7, repectively).

Overall, the G6PD deficiency affected more males than females, being 5.7% and 1.6, respectively. This is also verified in the number of males tested that were Intermediate or deficient (28 and 22 respectively), higher than the female (18 and 7). The opposite is verified in the samples normal, 35 in female and 11 in males. Doing the analysis of the G6PD deficient cases, the result is presented in the Table 3.

**Table 3:** Distribution of G6PD enzyme activity, analysed by spectrophotometry, according to sex.

G6PD Activity	Number	Sex	
		Male n (%)	Female n (%)
**≤ 10%**	9	8 (88.9)	1 (11.1)
**> 10–30%**	25	16 (64.0)	9 (36.9)
**> 30–60%**	27	17 (63.0)	10 (37.0)
**> 60%**	58	18 (31.0)	40 (69.0)
**Total**	**119**	**59 (49.6)**	**60 (50.4)**

For the screening test and taking into account its distribution of individuals by enzymatic activity, in according with WHO classification [25–27], it was verified that 0% (0/119) of the individuals were classified as Class I; 10.1% (12/119) of the individuals were classified as Class II; 48,7% (58/119) of the individuals were classified as Class III; 16.0% (19/119) of the individuals were classified as Class IV; and 25.2% (30/119) of the individuals were classified as Class V (Table 4).

**Table 4:** Classification of G6PD deficiency by sex in Cabo Verde in according with the WHO.

Classes	Enzimatic Value	Male	Female	Total	%	Enzymatic activity
		n	n	n		
I	< 0,12 U/gHg	0	0	0	0.00	associated with chronic non-spherocytic haemolytic anaemia (CNSHA); <1% residual activity
II	0,13–1,2 U/gHg	10	2	12	10.1%	severely deficient: 1–10% residual activity.
III	1,3–7,1 U/gHg	37	21	58	48.7%	moderately deficient: 10–60% residual activity
IV	7,2–17,7 U/gHg	9	10	19	16.0%	normal activity: 60–150%.
V	>17,7 U/gHg	3	27	30	25.2%	increased activity: 150% residual activity.

**Source:** Adapted by WHO, 1989 [25] and Kim et al., 2011 [27].

## Discussion

Malaria is considered as the greatest source of selective pressure known in mankind's recent history. The presence and the distribution of various polymorphisms associated with the surface antigens of the erythrocytes, including the globin genes (HbS, HbC, HbE) Thalassemias, oxidative stress (G6PD), cytoadherence and the immune system have been associated with a certain level of a protective effect against malaria [28].

G6PDd is considered one of the most common genetic disorders worldwide, but prevalence estimates are highest in Africa, Asia, the Mediterranean, and the Middle East [16, 29–31]. One of the main clinical consequences of G6PD deficiency, especially in new-borns is neonatal hyperbilirubinemia, which requires immediate diagnosis and treatment to prevent brain damage induced by irreversible bilirubin, known as kernicterus [32–34]. Furthermore, the use of certain anti-malarial drugs, including primaquine increase the risk of haemolysis for G6PD deficient patients [8]. Therefore, the WHO recommends the screening of the target population, and in the case of the presence of G6PDd among male with prevalence greater than 3% to ensure the treatment of jaundice [35].

In Cape Verde, the malaria treatment protocol relies on the WHO guidelines for the low-transmission area [6, 35]. Therefore, to reduce the transmission, single dose primaquine (0.25 mg/kg bw) together with an ACT is administrated to all patients with *P*. *falciparum* malaria, excepted for pregnant women, women breastfeeding a child under 6 months of age and children under 6 months of age. With the use of primaquine, it is crucial to evaluate the prevalence of G6PDd, especially in the current context of the ongoing malaria elimination programme which requires better management of all confirmed malaria cases to stop transmission. To our knowledge, this study is the first at national level, undertaken to provide such information to support the National Malaria Control Programme to achieve the elimination goal by implementing evidence-based control interventions. Indeed, despite being preliminary and partial the results reported here provide important data on the current prevalence of the G6PDd in Cape Verde. This is especially crucial in the islands with ongoing indigenous malaria and where primaquine is used to stop the transmission. Our study has shown the presence of the G6PDd, thus providing updated data for evidence-based intervention in the areas under consideration for better management of the use of primaquine to prevent its adverse effects.

This study determined the prevalence of total and partial G6PD deficiency among malaria affected and non-affected groups in Cape Verde. Among the Cape Verdean study population, the overall prevalence of G6PDd was 2.5%. This value is more than three times higher in males (5.7%) than in females (1.6%). These results are consistent with results from a preliminary study in the country in 2010 [36], and similary to the reports from other studies conducted in other African countries, as Mauritania [37], Uganda [38], Mozambique [39] Senegal [40,41] and Gambia [42]. The high G6PDd prevalence in the African or Afro-descendant population as shown by several studies [39–43] may reflect the population's exposure to malaria endemicity or ethnicity related [8–16]. However, our results do not allow drawing any conclusions of this nature since almost all the participants of this study were of Cape Verdean citizenship and no ethnic classification was made. Furthermore, the Cape Verdean population genetic background may have been more or less homogenized by a lot of miscegenation.

Our results showed that G6PDd affects more males than females in the country. In fact, the locus of the G6PDd recessive gene has been mapped into the X chromosome. Consequently, a male with the mutation will be hemizygote and will have full expression of the gene causing G6PD deficiency [44]. In contrast, a female may be either heterozygous or rarely homozygous for G6PD deficiency [45].

Studies demonstrate that the distributions of G6PD deficient in males is predominantly lie under the 30% G6PD activity thresholds and heterozygous females contribute predominantly to the intermediate activity ranges of less than 80% activity [46]. Our results revealed majority of the intermediate was in male, different from the expected result, what could be related with the performance Care-Start test kit [46,47]. Hence the need for other studies with quantitative tests, in the country, to address the gender disparity, once that, qualitative tests misclassify females with intermediate G6PD activity as normal [48]. In the previous study conducted in the country [36], about measuring of G6PD genotype, revealed also a very low frequency of G6PD deficiency associated alleles (A− and MED).

Several studies have also suggested that G6PD has a protective effect against *P*. *falciparum* malaria [30,49], with few studies related to *P*. *vivax*. Additionally, recent studies in West African countries [16, 39, 50], suggests that the diversity of common G6PD deficiency alleles in parts of West Africa is probably greater than that considered previously. In addition, a study carried out in the Gambia [32] investigating multiple G6PD deficiency alleles concluded that such alleles were associated with protection from severe malaria overall but exhibited differential effects with respect to two important clinical presentations of severe malaria, conferring protection from cerebral malaria and increased risk for severe malarial anaemia. Another study [21] to assess the presence and the extent of a protective association between G6PD deficiency and malaria, detected the absence of a negative association between G6PD deficiency not only with uncomplicated *P*. *falciparum* malaria but also with severe malaria. Similarly, the same study concluded, no significant association between G6PD deficiency and other species of malaria, including *P*. *vivax* and *P*. *malariae* or combinations of any two or all the three species, and not evident conclusion on the exact relationship. Another conclusion is that G6PDd is endemic in Africa, and the African G6PDd patients have relatively higher enzyme activity and milder consequences than Mediterranean or Asian, which could be one possible explanation the observed G6PD associated protect from *P*. *falciparum* malaria in Africa [21, 28].

The WHO (1989) [25] classifies G6PD deficiency according to the level of G6PD enzyme activity with Class I is associated with chronic non-spherocytic haemolytic anaemia (CNSHA); Class II as severely deficient with less than 10% residual activity; Class III being moderately deficient with 10±60% residual activity; Class IV showing normal activity of 60±150%; and Class V being increased activity. Adverse events are generally confined to individuals with G6PD activity < 10% of normal (WHO Class II). The common African variant G6PD A usually causes a mild to moderate deficiency while the MED variant is more severe.

Although in some study using the CareStart^™^ G6PD rapid test [51–53], had been demonstrated to have high level of specificity, in our study specificty was low. However, these results are similar to those found in other regions, namely Brazil, Cambodia or Ghana, particularly, where the test reported low sensitivity among individuals with enzyme activity. In the same time, the high number of false deficient RDT results and in comparison with the reference method (spectrophotometry) could be associate with the low sensitivity of the spectrophotometry method used in the identifying G6PDd individuals, as reported in recent study [54].

Some studies report inconsistency in the definition of normal G6PD activity as well as the definition of test sensitivity and specificity used, what can justify the difference in the classification of our qualitative results [46, 53]. Others factors can be associated, including the biological conditions such as concomitant haemoglobinopathies, the storage conditions for whole blood and temperature [55, 56]. And the fact that the study be carried out in an archipelagic country, with samples collection on different islands, their conditioning and sending may have affected the results. Hence, given these preliminary results, comparative studies with others quantitative assays, techniques and methodologies are strongly recommended in the country, in order to complement these findings.

## Conclusion

This study carried out in Cabo Verde to estimate and quantify the prevalence of G6PD deficiency, concluded that, despite relatively low, the presence the G6PD deficiency, a greatest concern in an area where primaquine is used for malaria treatment in the ongoing elimination programme. The evidence reported here provide imperative information to the NMCP. However, data are partial thus complementary nationwide studies are necessary to map the distribution of the G6PDd across the archipelago, to perform the techinique and test used and to better support decision-making in malaria case management in the context of elimination in Cabo Verde.

## Supporting information

S1 Appendix The list of test results, RDT and spectrophotometry. (DOCX)

S1 Table (PDF)
