# Supplementary material for: The prevalence of glucose-6-phosphate dehydrogenase deficiency in the Cape Verdean population in the context of malaria elimination
Source: PLoS One. 2020 Mar 16;15(3):e0229574. doi: 10.1371/journal.pone.0229574 (PMC7075545; doi:10.1371/journal.pone.0229574)
Supplement: S1 Appendix — (DOCX) [file pone.0229574.s001.docx]

Supporting Information files

| Code | Municipality | Island | Sexe | Age | TDR Carestart | Quantification of G6PDd IH /g Hg | Values Enzyme G6PDd (IU H / gHg) |
| --- | --- | --- | --- | --- | --- | --- | --- |
| 001 | Praia | Santiago | Male | 56 | Positive | Intermediate | 7 |
| 002 | Praia | Santiago | Male | 13 | Positive | Intermediate | 7 |
| 003 | Praia | Santiago | Male | 28 | Positive | Deficient | 2 |
| 004 | Praia | Santiago | Male | 36 | Positive | Intermediate | 7 |
| 005 | Praia | Santiago | Female | 35 | Positive | Normal | 6 |
| 006 | Praia | Santiago | Male | 43 | Positive | Intermediate | 6 |
| 007 | Praia | Santiago | Female | 39 | Positive | Normal | 40 |
| 008 | Praia | Santiago | Female | 56 | Positive | Intermediate | 3 |
| 009 | Praia | Santiago | Female | 27 | Positive | Intermediate | 3 |
| 010 | Praia | Santiago | Female | 24 | Positive | Deficient | 2 |
| 011 | Praia | Santiago | Female | 42 | Positive | Normal | 18 |
| 012 | Praia | Santiago | Male | 13 | Positive | Intermediate | 3 |
| 013 | Praia | Santiago | Female | 43 | Positive | Intermediate | 7 |
| 014 | Praia | Santiago | Female | 70 | Positive | Deficient | 1 |
| 015 | Praia | Santiago | Male | 12 | Positive | Deficient | 1 |
| 016 | Praia | Santiago | Male | 27 | Positive | Deficient | 1 |
| 017 | Praia | Santiago | Male | 44 | Positive | Intermediate | 3 |
| 018 | Praia | Santiago | Female | 73 | Positive | Normal | 9 |
| 019 | Praia | Santiago | Female | 10 | Positive | Normal | 7 |
| 020 | Praia | Santiago | Male | 44 | Positive | Normal | 11 |
| 021 | Praia | Santiago | Female | 19 | Positive | Normal | 14 |
| 022 | Praia | Santiago | Male | 75 | Positive | Normal | 9 |
| 023 | Praia | Santiago | Male | 5 | Positive | Intermediate | 5 |
| 024 | Praia | Santiago | Male | 30 | Positive | Intermediate | 3 |
| 025 | Praia | Santiago | Male | 42 | Positive | Intermediate | 6 |
| 026 | Praia | Santiago | Male | 25 | Positive | Normal | 14 |
| 027 | Praia | Santiago | Male | 30 | Positive | Deficient | 1 |
| 028 | Praia | Santiago | Male | 13 | Positive | Normal | 12 |
| 029 | Praia | Santiago | Female | 33 | Positive | Normal | 8 |
| 030 | Praia | Santiago | Female | 67 | Positive | Normal | 14 |
| 031 | Praia | Santiago | Female | 33 | Positive | Normal | 12 |
| 032 | Praia | Santiago | Female | 28 | Positive | Normal | 7 |
| 033 | Praia | Santiago | Female | 61 | Positive | Normal | 9 |
| 034 | Praia | Santiago | Female | 23 | Positive | Intermediate | 6 |
| 035 | Praia | Santiago | Male | 26 | Positive | Intermediate | 6 |
| 036 | Praia | Santiago | Male | 29 | Positive | Normal | 13 |
| 037 | Praia | Santiago | Female | 62 | Positive | Intermediate | 5 |
| 038 | Praia | Santiago | Male | 53 | Positive | Deficient | 2 |
| 039 | Praia | Santiago | Male | 53 | Positive | Intermediate | 5 |
| 040 | Praia | Santiago | Female | 31 | Positive | Deficient | 2 |
| 041 | São Vicente | São Vicente | Female | 51 | Positive | Normal | 13 |
| 042 | São Vicente | São Vicente | Female | 39 | Positive | Deficient | 2 |
| 043 | são Vicente | São Vicente | Male | 46 | Positive | Deficient | 2 |
| 044 | São Vicente | São Vicente | Male | 38 | Positive | Deficient | 1 |
| 045 | São Vicente | São Vicente | Female | 59 | Positive | Normal | 10 |
| 046 | São Vicente | São Vicente | Female | 14 | Positive | Deficient | 1 |
| 047 | São Vicente | São Vicente | Female | 52 | Positive | Normal | 12 |
| 048 | São Vicente | São Vicente | Male | 48 | Positive | Deficient | 0 |
| 049 | São Vicente | São Vicente | Male | 33 | Positive | Intermediate | 5 |
| 050 | São Vicente | São Vicente | Male | 76 | Positive | Intermediate | 3 |
| 051 | São Vicente | São Vicente | Male | 55 | Positive | Deficient | 2 |
| 052 | São Vicente | São Vicente | Male | 74 | Positive | Deficient | 0 |
| 053 | São Vicente | São Vicente | Male | 64 | Positive | Intermediate | 5 |
| 054 | São Vicente | São Vicente | Female | 74 | Positive | Intermediate | 8 |
| 055 | São Vicente | São Vicente | Male | 55 | Positive | Deficient | 0 |
| 056 | São Vicente | São Vicente | Female | 51 | Positive | Intermediate | 6 |
| 057 | São Vicente | São Vicente | Female | 38 | Positive | Intermediate | 5 |
| 058 | São Vicente | São Vicente | Female | 35 | Positive | Intermediate | 5 |
| 059 | São Vicente | São Vicente | Male | 46 | Positive | Deficient | 2 |
| 060 | São Vicente | São Vicente | Male | 17 | Positive | Deficient | 0 |
| 061 | São Vicente | São Vicente | Female | 29 | Positive | Deficient | 2 |
| 062 | São Vicente | São Vicente | Female | 28 | Positive | Deficient | 2 |
| 063 | São Vicente | São Vicente | Male | 53 | Positive | Deficient | 2 |
| 064 | São Vicente | São Vicente | Male | 8 | Positive | Deficient | 2 |
| 065 | São Vicente | São Vicente | Male | 46 | Positive | Intermediate | 4 |
| 066 | São Vicente | São Vicente | Male | 47 | Positive | Deficient | 2 |
| 067 | Santa Cruz | Santiago | Male | 16 | Positive | Normal | 9 |
| 068 | Santa Cruz | Santiago | Female | 46 | Positive | Intermediate | 3 |
| 069 | Santa Catarina | Santiago | Male | 20 | Positive | Deficient | 2 |
| 070 | Santa Catarina | Santiago | Male | 17 | Positive | Intermediate | 5 |
| 071 | Santa Catarina | Santiago | Female | 39 | Positive | Normal | 14 |
| 072 | Santa Catarina | Santiago | Male | 12 | Positive | Normal | 10 |
| 073 | Santa Catarina | Santiago | Male | 12 | Positive | Deficient | 2 |
| 074 | Santa Catarina | Santiago | Female | 13 | Positive | Normal | 2 |
| 075 | Santa Catarina | Santiago | Female | 37 | Positive | Normal | 4 |
| 076 | Santa Catarina | Santiago | Male | 23 | Positive | Intermediate | 7 |
| 077 | Santa Catarina | Santiago | Male | 12 | Positive | Intermediate | 6 |
| 078 | São Felipe | Fogo | Female | 30 | Positive | Normal | 9 |
| 079 | São Felipe | Fogo | Male | 53 | Positive | Intermediate | 5 |
| 080 | São Felipe | Fogo | Male | 54 | Positive | Intermediate | 4 |
| 081 | São Felipe | Fogo | Male | 38 | Positive | Normal | 14 |
| 082 | São Felipe | Fogo | Female | 20 | Positive | Normal | 4 |
| 083 | São Felipe | Fogo | Female | 60 | Positive | Normal | 43 |
| 084 | São Felipe | Fogo | Male | 28 | Positive | Normal | 12 |
| 085 | São Felipe | Fogo | Female | 8 | Positive | Normal | 22 |
| 086 | São Felipe | Fogo | Male | 5 | Positive | Normal | 10 |
| 087 | São Felipe | Fogo | Female | 37 | Positive | Intermediate | 5 |
| 088 | São Felipe | Fogo | Female | 8 | Positive | Normal | 22 |
| 089 | São Felipe | Fogo | Male | 4 | Positive | Deficient | 2 |
| 090 | São Felipe | Fogo | Female | 13 | Positive | Normal | 8 |
| 091 | São Felipe | Fogo | Female | 66 | Positive | Intermediate | 3 |
| 092 | São Felipe | Fogo | Male | 13 | Positive | Intermediate | 6 |
| 093 | São Felipe | Fogo | Female | 22 | Positive | Intermediate | 7 |
| 094 | São Felipe | Fogo | Female | 49 | Positive | Intermediate | 3 |
| 095 | São Felipe | Fogo | Female | 35 | Positive | Normal | 5 |
| 096 | São Felipe | Fogo | Female | 30 | Positive | Normal | 11 |
| 097 | São Felipe | Fogo | Female | 37 | Positive | Intermediate | 8 |
| 098 | São Felipe | Fogo | Male | 35 | Positive | Deficient | 1 |
| 099 | São Felipe | Fogo | Female | 33 | Positive | Normal | 14 |
| 100 | São Felipe | Fogo | Female | 42 | Positive | Normal | 23 |
| 101 | São Felipe | Fogo | Female | 52 | Positive | Normal | 12 |
| 102 | São Felipe | Fogo | Male | 65 | Positive | Deficient | 1 |
| 103 | São Felipe | Fogo | Male | 34 | Positive | Deficient | 2 |
| 104 | São Felipe | Fogo | Male | 22 | Positive | Intermediate | 3 |
| 105 | São Felipe | Fogo | Female | 56 | Positive | Normal | 15 |
| 106 | São Felipe | Fogo | Female | 56 | Positive | Normal | 10 |
| 107 | São Felipe | Fogo | Male | 32 | Positive | Intermediate | 4 |
| 108 | São Felipe | Fogo | Male | 56 | Positive | Intermediate | 4 |
| 109 | Tarrafal | Santiago | Female | 32 | Positive | Normal | 50 |
| 110 | Tarrafal | Santiago | Female | 78 | Positive | Intermediate | 4 |
| 111 | Tarrafal | Santiago | Female | 54 | Positive | Intermediate | 5 |
| 112 | São Miguel | Santiago | Male | 42 | Positive | Intermediate | 5 |
| 113 | São Miguel | Santiago | Male | 15 | Positive | Intermediate | 6 |
| 114 | São Miguel | Santiago | Male | 32 | Positive | Normal | 1 |
| 115 | São Miguel | Santiago | Female | 17 | Positive | Normal | 8 |
| 116 | São Miguel | Santiago | Female | 17 | Positive | Normal | 4 |
| 117 | São Miguel | Santiago | Female | 32 | Positive | Intermediate | 4 |
| 118 | São Miguel | Santiago | Female | 40 | Positive | Normal | 26 |
| 119 | São Miguel | Santiago | Female | 27 | Positive | Normal | 20 |
