# Supplementary material for: The prevalence of glucose-6-phosphate dehydrogenase deficiency in the Cape Verdean population in the context of malaria elimination
Source: PLoS One. 2020 Mar 16;15(3):e0229574. doi: 10.1371/journal.pone.0229574 (PMC7075545; doi:10.1371/journal.pone.0229574)
Supplement: S1 Table — (PDF) [file pone.0229574.s002.pdf]

Code	Municipality	Island	Sex	Age	TDR Carestart Results	Quantification of G6PDd IU / gHb	Values Enzyme G6PDd (IU / gHb)
**PR109**	Praia	Santiago	Male	56	Positive	Intermediate	7,3
**PR152**	Praia	Santiago	Male	13	Positive	Intermediate	6,5
**PR159**	Praia	Santiago	Male	28	Positive	Deficient	1,7
**PR175**	Praia	Santiago	Male	36	Positive	Intermediate	6,9
**PR178**	Praia	Santiago	Female	35	Positive	Normal	59,9
**PR182**	Praia	Santiago	Male	43	Positive	Intermediate	6,2
**PR183**	Praia	Santiago	Female	39	Positive	Normal	40,3
**PR301**	Praia	Santiago	Female	56	Positive	Intermediate	3,2
**PR385**	Praia	Santiago	Female	27	Positive	Intermediate	2,8
**PR398**	Praia	Santiago	Female	24	Positive	Deficient	1,5
**PR415**	Praia	Santiago	Female	42	Positive	Normal	18,3
**PR442**	Praia	Santiago	Male	13	Positive	Intermediate	3,2
**PR444**	Praia	Santiago	Female	43	Positive	Intermediate	7,1
**PR495**	Praia	Santiago	Female	70	Positive	Deficient	0,6
**PR675**	Praia	Santiago	Male	12	Positive	Deficient	1,1
**PR681**	Praia	Santiago	Male	27	Positive	Deficient	0,9
**PR703**	Praia	Santiago	Male	44	Positive	Intermediate	2,8
**PR719**	Praia	Santiago	Female	73	Positive	Normal	85,1
**PR723**	Praia	Santiago	Female	10	Positive	Normal	72,8
**PR750**	Praia	Santiago	Male	44	Positive	Normal	106,6
**PR755**	Praia	Santiago	Female	19	Positive	Normal	142
**PR842**	Praia	Santiago	Male	75	Positive	Normal	8,8
**PR894**	Praia	Santiago	Male	5	Positive	Intermediate	5
**PR902**	Praia	Santiago	Male	30	Positive	Intermediate	3,2
**PR923**	Praia	Santiago	Male	42	Positive	Intermediate	5,8
**PR927**	Praia	Santiago	Male	25	Positive	Normal	14
**PR1199**	Praia	Santiago	Male	30	Positive	Deficient	0,6
**PR1268**	Praia	Santiago	Male	13	Positive	Normal	119,6
**PR1332**	Praia	Santiago	Female	33	Positive	Normal	81,6
**PR1380**	Praia	Santiago	Female	67	Positive	Normal	138,5
**PR1395**	Praia	Santiago	Female	33	Positive	Normal	12,3
**PR1537**	Praia	Santiago	Female	28	Positive	Normal	66,1
**PR1541**	Praia	Santiago	Female	61	Positive	Normal	86
**PR1573**	Praia	Santiago	Female	23	Positive	Intermediate	6,2
**PR1619**	Praia	Santiago	Male	26	Positive	Intermediate	6,2
**PR1621**	Praia	Santiago	Male	29	Positive	Normal	13,1
**PR1636**	Praia	Santiago	Female	62	Positive	Intermediate	5,4
**PR1639**	Praia	Santiago	Male	53	Positive	Deficient	1,9
**PR1789**	Praia	Santiago	Male	53	Positive	Intermediate	5,4
**PR1791**	Praia	Santiago	Female	31	Positive	Deficient	1,5

**SV032**	São Vicente	São Vicente	Female	51	Positive	Normal	128,2
**SV051**	São Vicente	São Vicente	Female	39	Positive	Deficient	1,5
**SV159**	São Vicente	São Vicente	Male	46	Positive	Deficient	1,5
**SV183**	São Vicente	São Vicente	Male	38	Positive	Deficient	0,6
**SV189**	São Vicente	São Vicente	Female	59	Positive	Normal	9,7
**SV192**	São Vicente	São Vicente	Female	14	Positive	Deficient	1,1
**SV193**	São Vicente	São Vicente	Female	52	Positive	Normal	12,3
**SV288**	São Vicente	São Vicente	Male	48	Positive	Deficient	0,2
**SV308**	São Vicente	São Vicente	Male	33	Positive	Intermediate	5
**SV324**	São Vicente	São Vicente	Male	76	Positive	Intermediate	3,2
**SV328**	São Vicente	São Vicente	Male	55	Positive	Deficient	1,5
**SV337**	São Vicente	São Vicente	Male	74	Positive	Deficient	0,2
**SV352**	São Vicente	São Vicente	Male	64	Positive	Intermediate	5
**SV367**	São Vicente	São Vicente	Female	74	Positive	Intermediate	7,5
**SV408**	São Vicente	São Vicente	Male	55	Positive	Deficient	0,2
**SV409**	São Vicente	São Vicente	Female	51	Positive	Intermediate	5,8
**SV438**	São Vicente	São Vicente	Female	38	Positive	Intermediate	5,4
**SV514**	São Vicente	São Vicente	Female	35	Positive	Intermediate	5,4
**SV544**	São Vicente	São Vicente	Male	46	Positive	Deficient	1,9
**SV737**	São Vicente	São Vicente	Male	17	Positive	Deficient	0,2
**SV819**	São Vicente	São Vicente	Female	29	Positive	Deficient	2,4
**SV851**	São Vicente	São Vicente	Female	28	Positive	Deficient	1,5
**SV871**	São Vicente	São Vicente	Male	53	Positive	Deficient	1,9

**SV936**	São Vicente	São Vicente	Male	8	Positive	Deficient	2,4
**SV954**	São Vicente	São Vicente	Male	46	Positive	Intermediate	4,1
**SV969**	São Vicente	São Vicente	Male	47	Positive	Deficient	1,5
**SCZ009**	Santa Cruz	Santiago	Male	16	Positive	Normal	9,3
**SCZ208**	Santa Cruz	Santiago	Female	46	Positive	Intermediate	2,8
**SC019**	Santa Catarina	Santiago	Male	20	Positive	Deficient	2,2
**SC086**	Santa Catarina	Santiago	Male	17	Positive	Intermediate	4,7
**SC150**	Santa Catarina	Santiago	Female	39	Positive	Normal	143,5
**SC247**	Santa Catarina	Santiago	Male	12	Positive	Normal	10,1
**SC248**	Santa Catarina	Santiago	Male	12	Positive	Deficient	1,5
**SC251**	Santa Catarina	Santiago	Female	13	Positive	Normal	24,3
**SC254**	Santa Catarina	Santiago	Female	37	Positive	Normal	41,1
**SC282**	Santa Catarina	Santiago	Male	23	Positive	Intermediate	7,1
**SC490**	Santa Catarina	Santiago	Male	12	Positive	Intermediate	6,2
**FG001**	São Felipe	Fogo	Female	30	Positive	Normal	8,6
**FG040**	São Felipe	Fogo	Male	53	Positive	Intermediate	4,5
**FG041**	São Felipe	Fogo	Male	54	Positive	Intermediate	3,9
**FG106**	São Felipe	Fogo	Male	38	Positive	Normal	137,4
**FG110**	São Felipe	Fogo	Female	20	Positive	Normal	40,9
**FG115**	São Felipe	Fogo	Female	60	Positive	Normal	429,5
**FG117**	São Felipe	Fogo	Male	28	Positive	Normal	11,6
**FG120**	São Felipe	Fogo	Female	8	Positive	Normal	22,2
**FG123**	São Felipe	Fogo	Male	5	Positive	Normal	9,5
**FG151**	São Felipe	Fogo	Female	37	Positive	Intermediate	4,6
**FG262**	São Felipe	Fogo	Female	8	Positive	Normal	22,2
**FG264**	São Felipe	Fogo	Male	4	Positive	Deficient	2,4
**FG276**	São Felipe	Fogo	Female	13	Positive	Normal	77,8
**FG336**	São Felipe	Fogo	Female	66	Positive	Intermediate	3,2
**FG341**	São Felipe	Fogo	Male	13	Positive	Intermediate	5,8
**FG346**	São Felipe	Fogo	Female	22	Positive	Intermediate	6,7
**FG380**	São Felipe	Fogo	Female	49	Positive	Intermediate	2,8
**FG383**	São Felipe	Fogo	Female	35	Positive	Normal	51,1
**FG396**	São Felipe	Fogo	Female	30	Positive	Normal	110,1

**FG415**	São Felipe	Fogo	Female	37	Positive	Intermediate	7,5
**FG425**	São Felipe	Fogo	Male	35	Positive	Deficient	0,6
**FG430**	São Felipe	Fogo	Female	33	Positive	Normal	13,6
**FG440**	São Felipe	Fogo	Female	42	Positive	Normal	23
**FG442**	São Felipe	Fogo	Female	52	Positive	Normal	12,3
**FG444**	São Felipe	Fogo	Male	65	Positive	Deficient	1,1
**FG502**	São Felipe	Fogo	Male	34	Positive	Deficient	1,5
**FG528**	São Felipe	Fogo	Male	22	Positive	Intermediate	2,8
**FG541**	São Felipe	Fogo	Female	56	Positive	Normal	14,9
**FG542**	São Felipe	Fogo	Female	56	Positive	Normal	10,1
**FG548**	São Felipe	Fogo	Male	32	Positive	Intermediate	3,7
**FG560**	São Felipe	Fogo	Male	56	Positive	Intermediate	3,7
**T090**	Tarrafal	Santiago	Female	32	Positive	Normal	50
**T152**	Tarrafal	Santiago	Female	78	Positive	Intermediate	4,1
**T176**	Tarrafal	Santiago	Female	54	Positive	Intermediate	4,5
**SM012**	São Miguel	Santiago	Male	42	Positive	Intermediate	4,7
**SM071**	São Miguel	Santiago	Male	15	Positive	Intermediate	5,6
**SM073**	São Miguel	Santiago	Male	32	Positive	Normal	14,2
**SM147**	São Miguel	Santiago	Female	17	Positive	Normal	79,1
**SM160**	São Miguel	Santiago	Female	17	Positive	Normal	36,8
**SM164**	São Miguel	Santiago	Female	32	Positive	Intermediate	3,7
**SM172**	São Miguel	Santiago	Female	40	Positive	Normal	25,6
**SM216**	São Miguel	Santiago	Female	27	Positive	Normal	19,6
